# Neural Response During a Mechanically Assisted Spinal Manipulation in an Animal Model: A Pilot Study

**DOI:** 10.17352/2455-5487.000021

**Published:** 2015-04-06

**Authors:** William R. Reed, Michael A.K. Liebschner, Randall S. Sozio, Joel G. Pickar, Maruti R. Gudavalli

**Affiliations:** 1 Palmer Center for Chiropractic Research, Davenport, IA, USA; 2 Department of Neurosurgery, Baylor College of Medicine, Houston, TX, USA; 3 Research Service Line, Michael E. DeBakey VA Medical Center, Houston, TX, USA; 4 Exponent Failure Analysis, Houston, TX, USA

**Keywords:** Spinal manipulation, Muscle spindle, Neurons afferent, Neurophysiology, Zygapophyseal joint, Spinal fixation, Manual therapy, Cat

## Abstract

**Introduction:**

Mechanoreceptor stimulation is theorized to contribute to the therapeutic efficacy of spinal manipulation. Use of mechanically-assisted spinal manipulation (MA-SM) devices is increasing among manual therapy clinicians worldwide. The purpose of this pilot study is to determine the feasibility of recording *in vivo* muscle spindle responses during a MA-SM in an intervertebral fixated animal model.

**Methods:**

Intervertebral fixation was created by inserting facet screws through the left L_5-6_ and L_6-7_ facet joints of a cat spine. Three L_6_muscle spindle afferents with receptive fields in back muscles were isolated. Recordings were made during MA-SM thrusts delivered to the L_7_ spinous process using an instrumented Activator IV clinical device.

**Results:**

Nine MA-SM thrusts were delivered with peak forces ranging from 68-122N and with thrust durations of less than 5ms. High frequency muscle spindle discharge occurred during MA-SM. Following the MA-SM, muscle spindle responses included returning to pre-manipulation levels, slightly decreasing for a short window of time, and greatly decreasing for more than 40s.

**Conclusion:**

This study demonstrates that recording *in vivo* muscle spindle response using clinical MA-SM devices in an animal model is feasible. Extremely short duration MA-SM thrusts (<5ms) can have an immediate and/or a prolonged (> 40s) effect on muscle spindle discharge. Greater peak forces during MA-SM thrusts may not necessarily yield greater muscle spindle responses. Determining peripheral response during and following spinal manipulation may be an important step in optimizing its’ clinical efficacy. Future studies may investigate the effect of thrust dosage and magnitude.

## Introduction

Spinal manipulation is a form of manual therapy commonly used by clinicians and therapists for conservative treatment of musculoskeletal complaints. Spinal manipulation is typically distinguished from spinal mobilization by the presence of a short duration mechanical thrust applied to the spinal column using either direct hand contact (≤150ms) or one of several commercially available mechanical devices (≤10ms) [1-4]. Among chiropractic clinicians, use of mechanically-assisted spinal manipulation (MA-SM) is growing rapidly with reports that 40-60% of practitioners in the United States, Britain, Belgium, Canada, Australia, and New Zealand use MA-SM in some capacity of patient care [5-10].

Spinal manipulation has been shown to be effective in the treatment of neck and low back pain and is recommended by clinical guidelines and evidence reports [11-16]. Several reviews regarding the clinical efficacy, safety, usage, and mechanical effects of MA-SM have recently been published [17-20]. A majority of the MA-SM reviews have noted study weaknesses such as small sample size, non-randomization, and/or lack of a placebo or control group. Despite these limitations, great strides have recently been made in determining the mechanical characteristics and/or biological effects of MA-SM [1-4,21-31]. These studies may provide a foundation for larger randomly controlled trials of MA-SM therapy. One distinct advantage MA-SM offers over manually delivered manipulative thrusts in a research setting is that the thrust velocity and thrust magnitude can be standardized. This feature is of particular importance in efficacy and mechanistic studies investigating the biomechanical and/or neurophysiological effects of spinal manipulation. In addition, MA-SM devices can be mechanically altered to provide an adequate sham spinal manipulation (no force delivered) which is more difficult to accomplish with manually delivered manipulative thrusts.

Spinal manipulation by its very nature is a mechanical stimulus typically applied at clinically identified sites of intervertebral joint fixation or joint hypomobility. Theorized mechanisms for its therapeutic effects include breaking of joint adhesions and/or alteration of sensory input from primary afferents of paraspinal tissues which subsequently act to influence spinal cord reflexes and/or other central neural mechanisms [32,33]. MA-SM has been shown to result in oscillatory intervertebral movements [4,24,29,34,35] and neurophysiological responses in the form of bilateral compound action potentials in both *in vivo* animal [24,36] and human [21,23,29] studies. The compound action potentials from spinal nerve roots have been attributed to the simultaneous activation of mechano-sensitive afferents innervating viscoelastic spinal tissues such as muscles, ligaments, facet joints, and discs, but the exact sources of neural activity were not identified [23,29,37]. Muscle spindles are likely among the mechanoreceptors stimulated by MA-SM. They provide the central nervous system with sensory information regarding both changes in muscle length and the velocity at which those length changes occur. Using a feedback motor control system, we have previously shown that manipulative thrust durations between 25 and 150ms elicit high frequency discharge from paraspinal muscle spindles [38-40]. However to our knowledge, recordings of muscle spindle response associated with short manipulative thrust durations (≤10ms) as generated with clinical MA-SM devices, have never been recorded. It is unclear whether the noise artifact or high frequency mechanical perturbation associated with use of short thrust duration MA-SM devices would prohibit, obscure, or otherwise interfere with dorsal root recordings in a cat preparation. Therefore, the primary goal of this pilot study was to determine the feasibility of recording primary afferent muscle spindle responses in dorsal rootlets using a commercially available MA-SM device in an *in vivo* feline model of intervertebral joint fixation.

## Materials and Methods

The experimental preparation and procedures used in this study have been described in greater detail elsewhere [39-42] and are therefore presented here only briefly. Electrophysiological recordings were made from 3 back muscle spindle afferents traveling in the dorsal roots of a single Nembutal-anesthetized (35 mg/kg, iv; Oak Pharmaceuticals, Lake Forest, IL) adult male cat (4.5 kg). All experimental procedures were approved by the Institutional Animal Care and Use Committee (#20120601). This pilot data using a MA-SM device was collected from an experimental preparation associated with a separate study investigating the relationship between intervertebral fixation and L_6_ spinal manipulation delivered by a computer controlled feedback motor.

**Figure 1:**
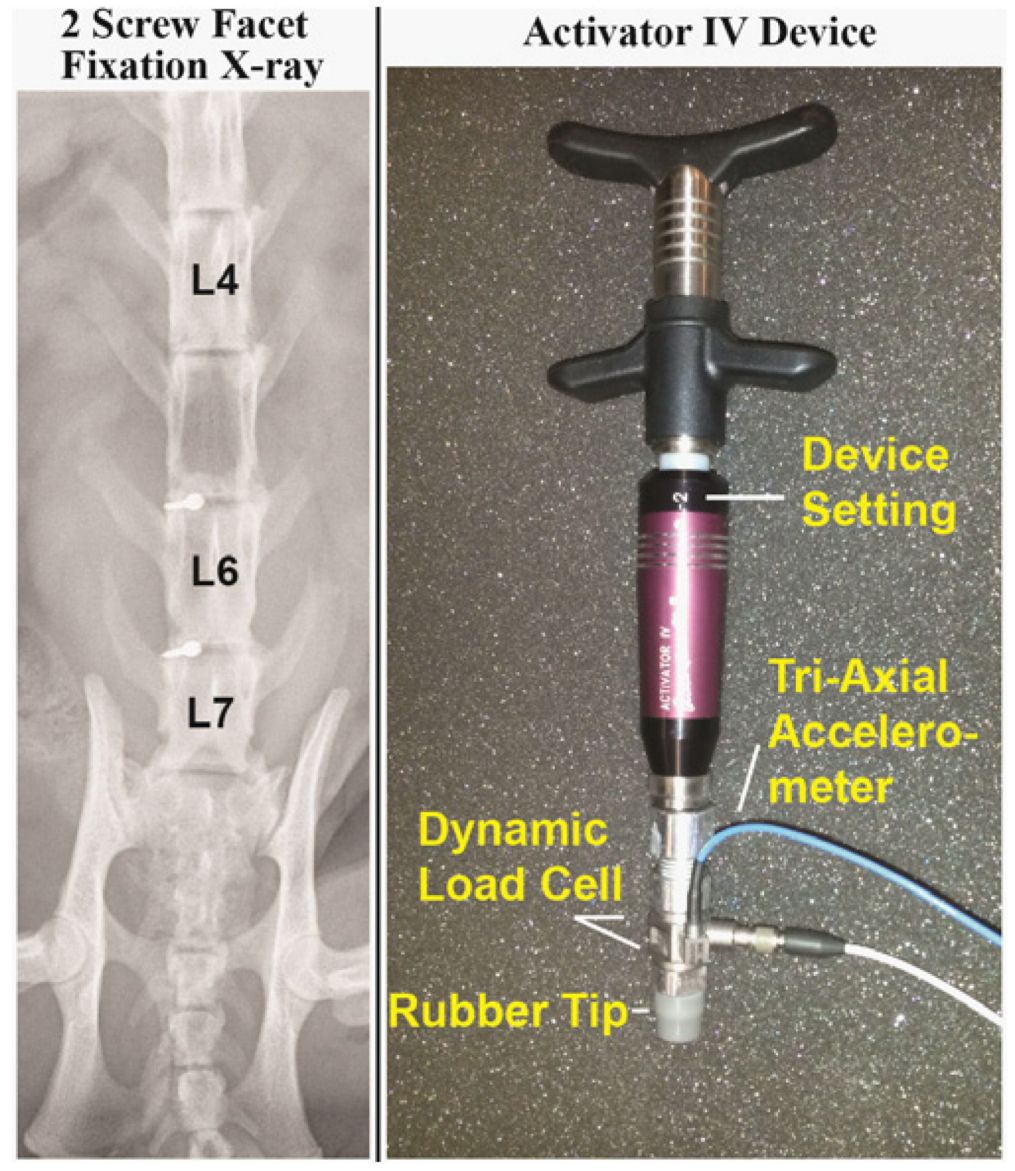
An x-ray of the unilateral L5-6 and L6-7 facet joint fixation and a photograph depicting the modified Activator IV device with attached dynamic load cell and tri-axial accelerometer.

Catheters were placed in the common carotid artery and external jugular vein to monitor blood pressure, introduce fluids and/or supplemental anesthesia if the arterial pressure rose above 120mm Hg or if a withdrawal reflex became present. The trachea was intubated and the cat was artificially ventilated. Since our focus was on back afferents, the right sciatic nerve was cut to reduce afferent input from the hindlimb. An L_5_ laminectomy was performed exposing the right L_6_ dorsal rootlets which were cut close to their entrance to the spinal cord and placed on a platform. Thin filaments were teased with fine forceps until action potentials from a single neuron were identified that responded to both mechanical pressure applied directly to the paraspinal back muscles (multifidus or longissimus) and a fast vibratory stimulus (~70 Hz; mini-therapeutic massage vibrator; North Coast Medical, Morgan Hill CA, USA). Afferent fibers remained positioned on the recording electrode while facet screws (10mm titanium endosteally anchored miniscrews; Dentaurum, Ispringen, Germany) were inserted through the left articular pillars of L_5-6_ and L_6-7_ vertebra in similar fashion to that previously described [40]. An x-ray of the L_5-6_ and L_6-7_ facet fixation is shown in Figure 1. Neural activity was passed through a high-impedance probe (HIP511, Grass, West Warwick, RI), amplified (P511 K, Grass) and recorded using a CED 1401 interface and Spike 2 data acquisition software (Cambridge Electronic Design, Cambridge, England).

### MA-SM Device

The Activator IV (Activator IV, Activator Methods Int. Ltd., Phoenix, AZ) is a hand-held clinical device comprised of a rubber-tipped spring-loaded hammer with 4 device settings that produce relative increases in thrust magnitude. Its thrust duration is<10ms and can deliver a maximum force of 212N when tested directly on a load cell [1]. For the current study, the device was modified by attaching an impedance head under the rubber tip (Figure 1). The impedance head included a dynamic load cell (Model 208C04; PCB, NY) and a tri-axial accelerometer (Model 356A01, PCB, NY).

Once a single back afferent had been isolated, the Activator IV device was placed by hand directly onto the exposed fascia overlying the cat’s L_7_ spinous process (one segment caudal to the level of afferent recording) and a small preload was applied. The L_7_vertebra was chosen to receive the MA-SM thrust due to the increased risk of tearing the L_6_ afferent fiber off the recording electrode during an L_6_ manipulation. The Activator IV device requires that a preload force be applied in order to completely retract the instrument tip prior to triggering the manipulative thrust. We used the two lowest device settings (1 and 2) which can deliver a force of 123N when tested directly on a load cell [1] but substantially less force (79N) when tested on polymer spinal tissue analog blocks [3]. MA-SM thrusts were applied in a dorsal-ventral direction and separated by a minimum period of 5 minutes. Electronic signals obtained from the force transducer and accelerometer were each sampled at 12,800 Hz and recorded in a binary file format on a computer using Lab View (National Instruments, Austin, TX).

## Results

Three muscle spindle afferents with receptive fields located in the longissimus back muscle were recorded during 9 L_7_ MA-SMs in a single cat preparation with intervertebral joint fixation. All afferents responded to mechanical movement of the spine and had sustained responses to fast vibratory stimuli (~70 Hz). All 3 afferents received MA-SM thrusts at a device setting of 1, whereas 2 afferents also received MA-SM thrusts at a device setting of 2. Individual MA-SM thrust profiles are reported in Table 1. All thrust durations were <5ms in duration and applied MA-SM peak forces ranged from 78.2 to 121.8N.

**Figure 2:**
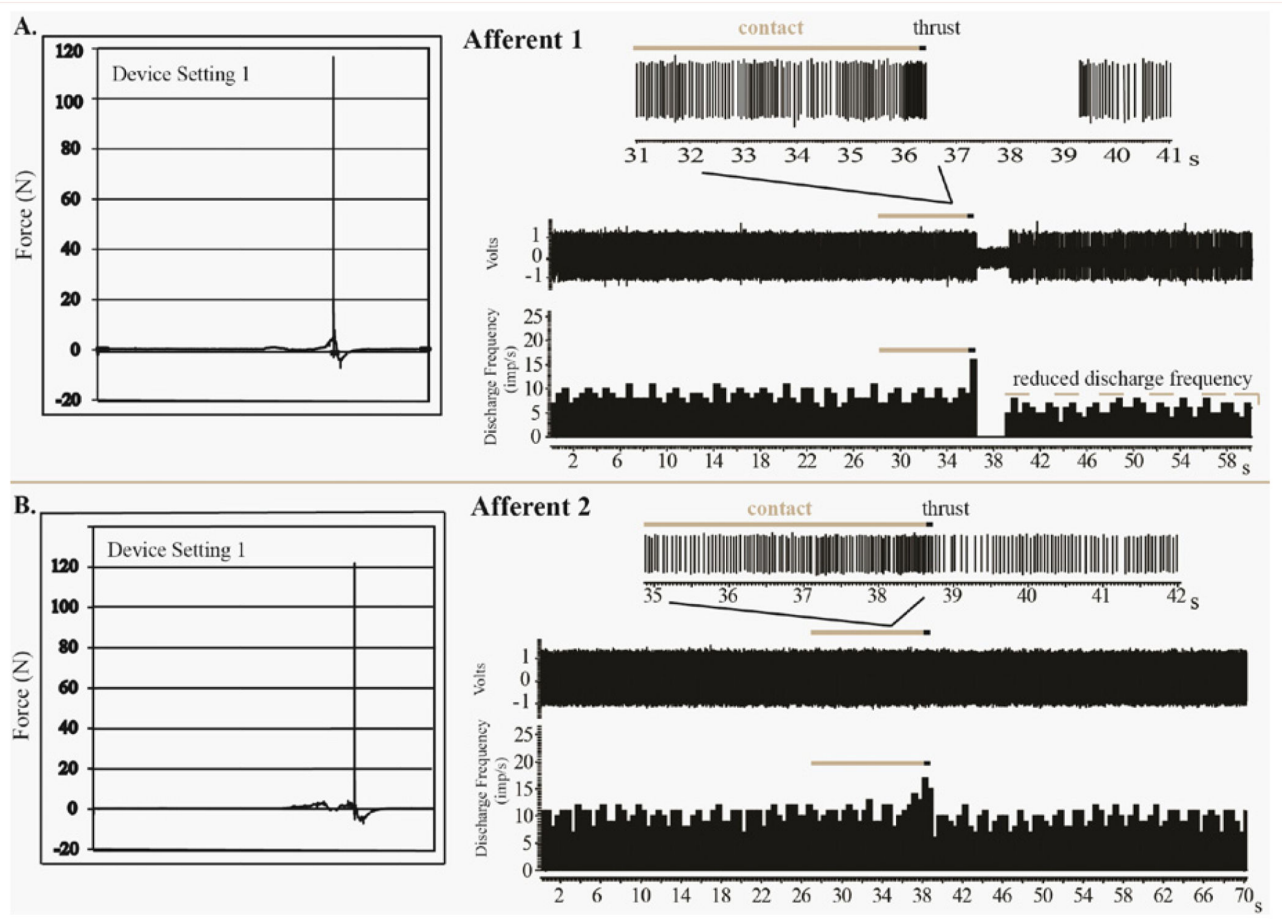
Recordings from 2 muscle spindle afferents in response to mechanically-assisted spinal manipulation (setting 1) with applied peak forces of 116.6N (A) and 121.8N (B). In Afferent 1, there was a 2.89s cessation of spindle discharge immediately following the manipulative thrust and slightly reduced resting discharge for at least 20s after the thrust. In Afferent 2, there was no cessation of discharge following the thrust and near immediate return of resting spindle discharge frequency despite similar peak thrust forces being delivered to the two afferents.

Examples of spindle responses to MA-SM thrusts from Afferent 1 and 2 are shown in Figure 2. For afferent 1 at a device setting of 1, the combined preload and MA-SM peak thrust force was 116.5N and the thrust duration was 2.0ms. The MA-SM thrust resulted in a high frequency spindle discharge during preload and thrust. Immediately following the thrust there was a 2.89s cessation of spindle discharge followed by the resumption of resting discharge but at a mean frequency slightly less than that prior to the thrust and lasting for the remaining 20s of recording (Figure 2A). For afferent 2 at a device setting of 1, the combined preload and peak MA-SM thrust force was 121.8N and the thrust duration was 2.0ms (Figure 2B, Table 1). Unlike Afferent 1, Afferent 2 exhibited no cessation of discharge following the MA-SM thrust and rapidly resumed resting discharge.

**Figure 3:**
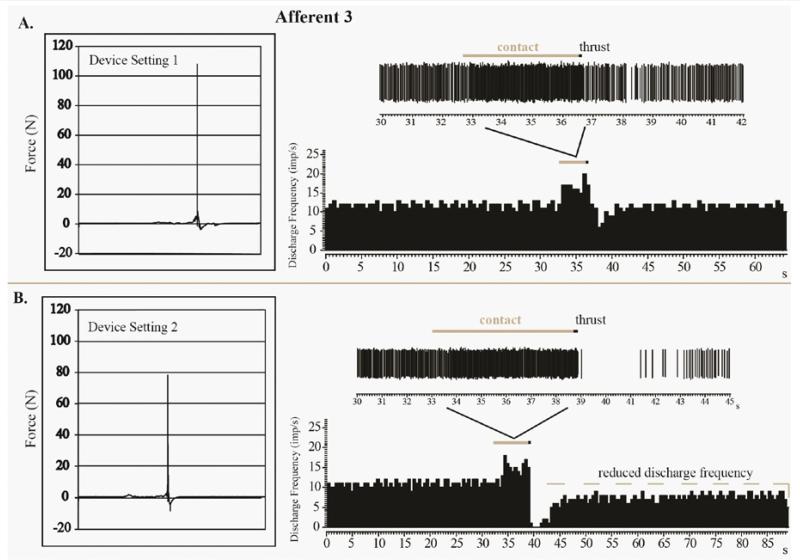
Recordings from a third muscle spindle afferent to mechanically-assisted spinal manipulations at device settings of 1 (A) and 2 (B). Greater peak forces were physically applied with setting 1 (107.9N) than with setting 2 (78.2N), however the lower total peak force produced an immediate and prolonged decrease in muscle spindle response following the manipulative thrust.

The four MA-SM thrusts using device setting 1 delivered to Afferent 3 had a mean peak force of 109N and mean thrust duration of 3.0ms (Table 1). Similar to Afferents 1 and 2, there was an increase of spindle discharge as a result of preload and MA-SM thrust at the L_7_ spinous process (Figure 3A). Following the thrust there was a decrease (but not a cessation) in spindle discharge lasting approximately 2.47s before there sumption of pre-thrust resting discharge frequency. For Afferent 3, mean peak force for the two MA-SM thrusts at device setting 2 was 81 N and mean thrust duration was 3.0ms. Afferent 3’s response to one of thrusts at device setting 2 is shown in Figure 3B. There was an increase in spindle discharge with preload and thrust similar to that when the device was set at 1. However, unlike with setting 1 post-thrust activity was further reduced and more prolonged (~4.13s) at device setting 2. Despite the lower peak force (78.2N) delivered on device setting 2 compared to device setting 1 (107.9 N), there is a prolonged period (>40s) during which resting discharge did not return to pre-thrust levels (Figure 3A, 3B). It should be noted that mean Afferent 3 resting discharge frequency prior to the MA-SM thrust delivered at device setting 1 or 2 were similar (Figure 3A,3B). Although the precise time is not known, Afferent 3 returned to its resting discharge frequency at some point within 5 min after the setting 2 thrust delivery depicted in Figure 3B. Afferent 3 also exhibited increased afferent discharge to a fast vibratory stimulus (70 Hz) after the thrust suggesting that no fiber damaged had occurred as a result of this MA-SM.

## Discussion

To our knowledge, this study is the first to record muscle spindle response evoked by a mechanically-assisted spinal manipulation device that is used in clinical practice. Because spinal manipulation is typically delivered at sites of clinically determined biomechanical joint dysfunction and/or pain provocation, the relationship between intervertebral joint fixation/hypomobility and sensory signaling elicited from paraspinal mechanoreceptors during spinal manipulation is of particular interest to manual therapy researchers and clinicians alike. The purpose of the facet fixation model was to produce a moderate degree of segmental dysfunction that might be similar to that encountered by manual therapy clinicians in practice. It will likely be through a combination of both basic and clinical research that the underlying physiological mechanisms of manual therapy will be elucidated and its clinical efficacy optimized.

Although this pilot study contained a limited number of afferents, it demonstrated some important findings and will help to inform future studies. First with regards to the preparation, we demonstrated the feasibility of recording muscle spindle responses in an *in vivo* animal model using a clinical MA-SM device. The afferent fiber was wrapped around the recording electrode and withstood the perturbation associated with the mechanical delivery of 78 to 122 N forces over extremely short durations (< 5ms). Evidence for a lack of damage to the afferent fiber is in part provided through the return of pre-thrust resting spindle discharge following MA-SM. The risk of potential afferent fiber damage during MA-SM delivery in this preparation is real, but can be minimized by using dorsal rootlets that are longer in length. Although noise artifacts were encountered during the experiments, this appeared due in large part to movement of the device while the operator delivered the thrust. This issue can be remedied by non-manually triggering the MA-SM device attached to a rigid frame or perhaps using newer electrically powered (non-spring-loaded) MA-SM devices [3].

**Figure 4:**
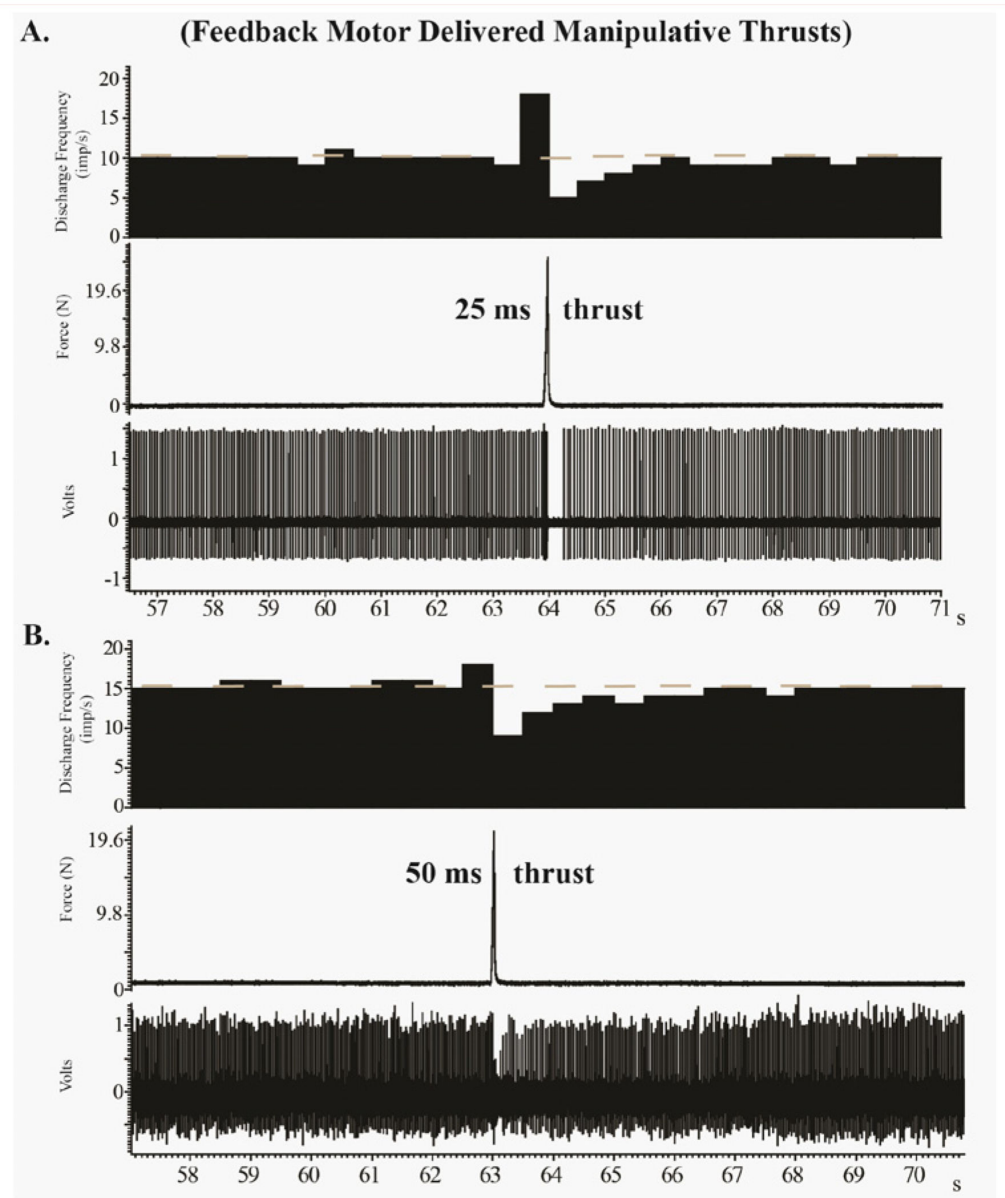
Recordings from two muscle spindle afferents in separate but similar cat experiments in which a mechanical feedback motor was used to deliver L_6_ manipulative thrusts of 25 ms (A) and 50 ms (B) duration without a tissue preload.In (A) there was a cessation of discharge (0.3 s) following a 24.5 N thrust, while in (B) there was a decrease in discharge (3.47 s) following a 19.6 N thrust. Cat body weight in (A) was 5.1 kg and in (B) 3.2 kg. Similarity in muscle spindle response characteristics between less forceful thrusts delivered by a feedback motor and greater forces delivered by the Activator IV device suggests a possible plateau effect for thrust magnitude on muscle spindle response.

We found that the extremely short MA-SM thrust durations elicited high frequency discharge from paraspinal muscle spindle afferents. This response appears similar that which occurs during 25-150ms thrust durations delivered by a computer-controlled feedback motor [38-40] (Figure 4), but direct comparisons are difficult due to the presence of preload forces and a lack of controlled preload durations in the current study. This pilot study clearly demonstrated that muscle spindle afferents can respond differently to similar MA-SM thrust forces (Figures 1-3). Afferents 1-3 exhibited post-thrust responses ranging from limited diminution of discharge (Afferent 2), to a mild decrease (Afferent 3) or complete cessation of discharge for nearly 3s (Afferent 1). It is not known, whether these differences in post-MA-SM thrust response are due to inherent differences related to muscle spindle intrafusal fiber types (e.g. bag vs chain fibers; for greater discussion in this regard see [43,44]), the anatomical proximity of the afferent’s receptive field to the L_7_ spinous process thrust site, and/or other biological factors. In a previous study investigating the effects of L_6_ and L_7_ anatomical thrust delivery sites on L_6_ muscle spindle discharge, we found that segmental contact sites distant to the muscle spindle’s receptive field were just as effective at increasing spindle discharge as contact sites close to the receptive field [45].

We found it interesting that the lower force delivered at setting 2 (78.2N) versus the higher peak force delivered at setting 1 force (107.9N) had a greater impact on Afferent 3’s discharge post-thrust (Figure 3). It is reasonable to think that greater forces delivered into the spine over the same duration would create greater vertebral displacement and thereby evoke a greater response from paraspinal muscle spindle afferents. However, several variables and conditions in the current experiment may affect this rationale including the use of extremely short thrust durations (<5 ms), a thrust site 1 segment caudal to afferent recording level, the presence of intervertebral fixation, and/or the greater inherent flexibility of the cat spine. Colloca and colleagues in a sheep model found that as the applied force increased vertebral displacements also increased [24,31]. However, they also found that a constant thrust force of 80N at L_3_ produced larger adjacent vertebral motions at shorter thrust durations (10ms) compared to longer thrust durations (100 and 200ms) [24,31]. It is thought that the mechanical principles of resonant frequency may apply to the human spine. If so, lower manipulative forces applied at resonance frequencies of the spine may accomplish similar vertebral motions as greater forces applied at nonresonant frequencies [17]. However, since settings 1 and 2 thrust durations are nearly equivalent, this particular explanation of differences in muscle spindle response is unlikely.

## Limitations

Preload forces and preload durations were not standardized in the current study as the Activator IV device was operated by hand as is performed clinically. Applied preload forces are required to retract the tip of Activator IV device, but we consciously attempted to limit the magnitude of applied preload forces since the preload duration was not standardized. We used thrust force magnitudes in our animal model that were the same or similar to those used in human studies in the human cervical spine. In humans, mean peak forces during manually applied cervical manipulation has been reported to be 118N [46]. Although direct circumference measurements were not performed in this study, the actual trunk size of adult male cats appears to be similar to the anatomical size of the human neck. While, we acknowledge that the thrust forces used in the current study were up to 2.7× the cat’s body weight we must also be mindful that the whole lumbar spine stiffness of the cat spine has been shown to be 2-7× less than that of human spines. Species differences in spinal stiffness have been clearly demonstrated in that unlike human cadaveric specimens, structural failure did not occur in the cadaveric cat spines with flexion/extension biomechanical testing [47]. Figure 4 demonstrates that much smaller forces (24.5 N and 19.6 N) have similar effects on paraspinal muscle spindle response suggesting a plateau effect of thrust magnitude. In addition, previous studies have indicated that Activator devices produce a maximum of 0.3 J of kinetic energy which is far below the energies required to produce tissue injury [36,48]. As is the case clinically, the Activator IV device is commonly used on much smaller human body parts than the human neck such as the wrists, elbows or ankles [49].

## Conclusion

This pilot study demonstrates feasibility of recording *in vivo* muscle spindle response during spinal manipulation using clinical mechanically-assisted spinal manipulation devices. It also demonstrates that extremely short duration manipulative thrusts (<5ms) of equivalent forces to that delivered to the human cervical spine can have an immediate and/or perhaps a prolonged effect (> 40s) on paraspinal muscle spindle discharge. While the clinical relevance of how mechanoreceptor stimulation or inhibition related to spinal manipulation modulates central nervous system activity remains to be clarified, determining how various mechanoreceptors respond during and following spinal manipulative thrusts in a clinically relevant fashion is an important step toward achieving this goal.

## Figures and Tables

**Table 1:** Mechanical-Assisted Spinal Manipulation Thrust Profiles The thrust profiles of mechanical-assisted spinal manipulation using the Activator IV instrumented device for the 3 muscle spindle afferents in this study are shown. Total peak force includes preload which can be influenced by the device operator.

Afferent Number	Device Setting	Thrust Duration (ms)	Preload Force (N)	Total Peak Force (N)
1	1	2.0	9.1	116.5
2	1	3.0	6.7	121.8
2	2	2.1	9.7	115.9
3	1	3.0	6.5	106.6
3	1	3.0	4.5	111.2
3	1	3.0	5.7	110.3
3	1	3.0	7.5	107.9
3	2	3.0	10.8	83.9
3	2	3.0	4.9	78.2
